# Improved risk stratification in prevention by use of a panel of selected circulating microRNAs

**DOI:** 10.1038/s41598-017-04040-w

**Published:** 2017-07-03

**Authors:** Till Keller, Jes-Niels Boeckel, Stefan Groß, Jens Klotsche, Lars Palapies, David Leistner, Lars Pieper, Günnter K. Stalla, Hendrik Lehnert, Sigmund Silber, David Pittrow, Winfried Maerz, Marcus Dörr, Hans-Ulrich Wittchen, Sebastian E. Baumeister, Uwe Völker, Stephan B. Felix, Stefanie Dimmeler, Andreas M. Zeiher

**Affiliations:** 1 Department of Internal Medicine III, Cardiology, University Hospital, Goethe University Frankfurt, Frankfurt, Germany; 2 German Centre for Cardiovascular Research (DZHK), Partner site RheinMain, Frankfurt, Germany; 3 Institute of Cardiovascular Regeneration, Centre for Molecular Medicine, Goethe University Frankfurt, Frankfurt, Germany; 4 Institute of Clinical Psychology and Psychotherapy, Technical University Dresden, Dresden, Germany; 5 Department of Internal Medicine B, University Medicine Greifswald, Greifswald, Germany; 6 Max Planck Institute of Psychiatry, Endocrinology, Munich, Munich, Germany; 7 University Clinic Schleswig-Holstein, Lübeck, Germany; 8 Outpatient Clinic, Munich, Germany; 9 Synlab Akademie für ärztliche Fortbildung, Synlab Services GmbH, Mannheim, Germany; 10 Interfaculty Institute for Genetics and Functional Genomics, Department of Cardiology, University Medicine Greifswald, Greifswald, Germany; 11 Institute of Clinical Pharmacology, Technical University Dresden, Dresden, Germany; 12 Institute for Community Medicine, University Medicine Greifswald, Greifswald, Germany; 13 Institute of Epidemiology and Preventive Medicine, University of Regensburg, Regensburg, Germany; 14 German Centre for Cardiovascular Research (DZHK), Partner site Berlin, Germany; 15 German Centre for Cardiovascular Research (DZHK), Partner site Greifswald, Germany; 16 Department of Cardiology, Kerckhoff Heart and Thorax Centre, Bad Nauheim, Germany

## Abstract

Risk stratification is crucial in prevention. Circulating microRNAs have been proposed as biomarkers in cardiovascular disease. Here a miR panel consisting of miRs related to different cardiovascular pathophysiologies, was evaluated to predict outcome in the context of prevention. MiR-34a, miR-223, miR-378, miR-499 and miR-133 were determined from peripheral blood by qPCR and combined to a risk panel. As derivation cohort, 178 individuals of the DETECT study, and as validation cohort, 129 individuals of the SHIP study were used in a case-control approach. Overall mortality and cardiovascular events were outcome measures. The Framingham Risk Score(FRS) and the SCORE system were applied as risk classification systems. The identified miR panel was significantly associated with mortality given by a hazard ratio(HR) of 3.0 (95% (CI): 1.09–8.43; p = 0.034) and of 2.9 (95% CI: 1.32–6.33; p = 0.008) after adjusting for the FRS in the derivation cohort. In a validation cohort the miR-panel had a HR of 1.31 (95% CI: 1.03–1.66; p = 0.03) and of 1.29 (95% CI: 1.02–1.64; p = 0.03) in a FRS/SCORE adjusted-model. A FRS/SCORE risk model was significantly improved to predict mortality by the miR panel with continuous net reclassification index of 0.42/0.49 (p = 0.014/0.005). The present miR panel of 5 circulating miRs is able to improve risk stratification in prevention with respect to mortality beyond the FRS or SCORE.

## Introduction

Ischemic heart disease is the major cause of death in western countries and is progressively occurring with increasing age^1^. Until the year 2050, the United Nations predict a constant decrease of the age group 25–49 years and, at the same time, an increase of individuals aged 50 years and above in developed countries^2^.

Given these facts, the need for new and improved preventive strategies arises. Use of an individualized therapy allows specific treatments of patients and may thereby be beneficial for the patient as well as for the healthcare system^3, 4^. Therefore, identification of patients suitable for specific therapies is of major importance. In primary care studies^5, 6^ as well as in clinical routine commonly scoring systems like the Framingham risk score (FRS)^7^ or the SCORE system of the European Society of Cardiology^8^ are used to identify patients at risk for overt cardiovascular events.

Recently, the use of various biomarkers has been proposed to facilitate such an identification processes. Several biomarkers are able to amend the prognostic ability of e.g. the FRS^9^. In this regard, it has been shown that elevated C-reactive protein (CRP) levels as biomarker for inflammation defines a patient population that benefits from statin therapy^3^. This underlines the potential of biomarkers as a useful tool e.g. in primary prevention. Furthermore, the use of a multiple biomarker approach, reflecting different pathophysiological aspects, has been shown to add useful prognostic information and, therefore, consistently improved risk estimation for incident cardiovascular event^9^.

MicroRNAs (miRs) are single-stranded, non-coding nucleotide sequences functioning by binding to specific target mRNAs and inhibiting their translational processing^10^. MiRs were detected in various body fluids and can also reliably be measured in peripheral blood^11^. Circulating miRs have been shown to be valid biomarkers in a broad spectrum of cardiovascular diseases such as acute myocardial infarction or stable heart disease^12, 13^. MiRs show specific regulation patterns in a transcript and disease related manner. This raises the question for an implementation of miRs as biomarkers for prevention on a multi-marker level.

Based on published literature and own work, several microRNAs seem promising to be included in such a multi-marker approach for the prediction of cardiovascular morbidity and mortality. MiR-34a was shown to regulate cardiac aging^14^. Furthermore, miR-34a is associated with left ventricular remodeling after myocardial function^14–16^. MiR-223 modulates inflammation, regulating granulocyte function^17^ and controlling cardiomyocyte glucose metabolism^18^.

MiR-378 counteracts obesity by controlling brown fat expansion^19^ and blocks cardiac hypertrophy^20^. MiR-133 modulates skeletal muscle proliferation and differentiation^20, 21^ and seems to control cardiac hypertrophy^22^.

Finally, the cardiac expressed miR-499, the prototype miR used as potential diagnostic biomarker indicating myocardial infarction^23^, that was suggested to also be a marker of myocyte damage in various cardiovascular diseases^24^.

The present study tests and validates the prognostic ability of these selected miRs for the prediction of cardiovascular outcome and death using a sample of primary care patients and an independent general population-sample. We further determined the potential amendatory prognostic information of circulating miRs within a multi marker approach on top of the established FRS or SCORE.

## Methods

### Study Population

For this investigation, data of two independent prospective cohorts were utilized. First, a derivation cohort was used to evaluate the prognostic value of the proposed miR panel in a low to intermediate risk real-world population representative for individuals presenting themselves to their primary care physician. Second, the predictive value of the miR panel was validated in a population-based sample excluding individuals with prevalent cardiovascular diseases to evaluate the use of the miR panel in the context of primary prevention. The local ethics review board approved the protocols of both studies an they were conducted in accordance with the Declaration of Helsinki. Written informed consent was obtained from each individual.

#### Derivation Cohort

As derivation cohort, data of the prospective longitudinal *Diabetes Cardiovascular Risk Evaluation Targets and Essential Data for Commitment of Treatment* (DETECT) study was use^25^. This study consists of a representative German sample of 3,188 primary care offices run by physicians including medical practitioners, general practitioners, and general internists. Here, 55,518 unselected consecutive individuals were enrolled on two predefined half-days. For laboratory analyses, a random sub-sample of 7,519 participants in 1,000 primary care practices was selected. Out of this sub-sample, 178 representative individuals were randomly chosen. This sample was used for the present analyses including 21 subjects who reached the pre-defined combined endpoint of occurrence of a cardiovascular event and overall mortality within a 5-year follow-up period and 157 controls. Cardiovascular event was defined as first occurrence of death from cardiovascular causes, non-fatal myocardial infarction or need for coronary revascularization including coronary bypass surgery and percutaneous coronary intervention. State of health and medical history within the follow-up period was obtained at the 5-year follow-up in 2008 from the primary care physician or by the institution in which the patients were previously treated using standardized assessment forms.

Venous blood samples were obtained at enrollment, immediately frozen and stored at −80 °C until further analyses. The DETECT study was approved by the ethics committee of the Carl Gustav Carus Medical Faculty, Technical University of Dresden (NCT01076608).

#### Validation Cohort

The Study of Health in Pomerania (SHIP) served as external validation cohort^26^. The SHIP is a population-based study that enrolled adult German residents of West Pomerania in northeastern Germany. For the present analyses, 64 individuals without a history of cardiovascular disease (CVD; including coronary artery disease, heart failure, history of stroke or peripheral artery disease) who died (death from any cause, primary endpoint) within a 12-year follow-up period were randomly chosen as cases. As controls, 65 additional individuals without prevalent CVD were selected from this study. Cases and controls were matched according to age and gender in a 1:1 ratio. As secondary outcome measure the combined endpoint of cardiovascular death, non-fatal myocardial infarction or stroke was used, reached by 43 out of the 129 individuals of the study cohort. Within the SHIP study a mortality follow-up is performed annually collecting information on vital status from population registries. Here, death certificates are obtained from the local health authorities. The underlying cause of death is validated independently by two medical doctors specialized in internal medicine. In case of disagreement a third internist is consulted. Further, a morbidity follow-up was obtained by postal questionnaires. Non- responders were contacted by telephone. Results were validated by the general practitioners^26^.

For all subjects, non-fasting blood samples were drawn, aliquoted and stored at −80 °C for further analysis. All study participants gave written informed consent. The SHIP study was approved by the ethics committee of the University of Greifswald.

### Laboratory Analyses

#### MicroRNA Panel

To test the hypothesis that a panel of distinct miRs is able to provide relevant prognostic information in prevention we selected 5 specific miRs. Selection was based on literature review of the authors as well as our own work. Criteria for selection were valid published data on an association with known cardiovascular risk factors or aspects of cardiovascular disease as well as the ability for quantification as circulating miRs in peripheral blood with respect to a potential use as biomarkers. Our selection included miR-34a with its role to regulate cardiac aging^14^, miR-223 in the context of inflammation^17, 18^, miR-378 associated with brown fat expansion^19^ and cardiac hypertrophy^20^, miR-133 with association to cardiac hypertrophy^22^, and the cardio-specific miR-499^24^. These microRNAs were quantified by qRT-PCR.

#### RNA Isolation and Quantification of miR levels

For isolation of total RNA, 250 μL EDTA-plasma was mixed with 700 µl TRIzol BD (Sigma; T3809), RNA was further isolated using the miRNeasy kit (Qiagen; 217004) using a Qiacube (Qiagen) sample preparation automat. The procedure was carried out in accordance to the manufacture’s protocol, with the exception that 30 µl of RNase-free purified water were used for final RNA elution.

RNA was then diluted 1:10 with H_2_O. The diluted RNA (5 μL) was reverse transcribed using the TaqMan microRNA Stemloop RT kit (ABI) according to the manufacture’s protocol. Subsequently, 3 μL of the product was used for detecting miR levels of miRs-34a, miR-223, miR-378, miR-499 and miR-133 by (q)PCR using TaqMan microRNA Assay kits (ABI) (Supplementary Methods). For qPCR using hydrolysis probes the VIAA7 Real-Time PCR System (ABI) was used. Relative miR levels were calculated as 2^−(CT[microRNA])^. For the current analyses, no normalization was done. RNA isolation and miR quantification were performed by experienced technicians blinded to the study participants clinical characteristics at a RNA core lab at the Goethe University Frankfurt.

#### Conventional Laboratory Analyses

Lipids and serum creatinine were measured at the respective study site using standardized laboratory methods. Glomerular filtration rate was estimated (eGFR) using the abbreviated *Modification of Diet in Renal Disease* (MDRD) formula. In the DETECT study, cardiac troponin I, NT-proBNP and CRP were quantified using commercially available assays with troponin I (Advia Centaur TnI- Ultra, Siemens), NT-proBNP and hsCRP (both Roche Diagnostics) and hsCRP. All measurements were carried out by experienced staff blinded to the participant’s characteristics.

### Statistical Analyses

Continuous variables are given as mean with corresponding standard deviation (SD) or as median with interquartile range in case of a skewed distribution. Associations between miRs and biomarkers such as laboratory parameters as well as clinical parameters were assessed with Spearman rank correlation coefficient. Survival functions were derived from a Kaplan-Meier estimator. To estimate the prognostic property of the miRs, Cox proportional hazards models for all-cause mortality or the combined cardiovascular endpoint were estimated. The miR measurements were taken as predictors. As the proportional hazards assumptions have been emphasized by the chi square statistic for Schoenfeld residuals, hazard ratios (HR) are given per standard deviation (SD) increase as well as after dichotomization with threshold based on an optimized Youden index. Wald confidence intervals and corresponding p-values are provided. HRs were calculated for an un-adjusted model as well as after adjustment for (i) age and sex and (ii) a combination of the Framingham risk score variables (age, gender, cholesterol, HDL, smoking, blood pressure). Along with the singular miRs, a score from a multivariate miR panel was calculated by means of an unadjusted logistic regression model with occurence of an event ((i) death and (ii) combined endpoint) as binary outcome: the setting of the model coefficients gives rise to a linear combination of predictors, such that its logit-transform is the risk estimated by the model, which defines the predicted score. To evaluate the additional prognostic information of the miR panel beyond a model based on a combination of the Framingham risk score variables receiver operator characteristic (ROC) analyses, Harrell’s c-index as well as reclassification indices have been used. Differences between respective area under the curve in the ROC analyses and between c-indices were calculated by the methods proposed by DeLong^27^ and Kang^28^. The continuous net reclassification index (NRI)^29^, and the absolute and relative integrated discrimination improvement (IDI)^30^ were calculated with a cNRI of 0.39 (0.61) considered as having relevant power^31^. All analyses were done using R 3.1.1 (R Foundation for Statistical Computing, Vienna, Austria).

## Results

### Characteristics of the derivation cohort

The derivation cohort comprised 178 representative individuals of the DETECT study, including 101 (56.7%) females, with median age of 55.5 (46–69) years. The characteristics of this study sub-cohort in comparison to the overall DETECT cohort are shown as Supplementary Table 1. Within the follow-up period of 5 years, 12 of these study participants died and 21 experienced the combined endpoint of a cardiovascular event or death. Table 1 provides the baseline characteristics of the derivation cohort classified by event state. As expected, individuals with poor outcome had a higher cardiovascular risk profile with respect to traditional risk factors such as arterial hypertension, dyslipidemia or diabetes (all p < 0.05) compared to event-free survivors. This is also depicted by a higher FRS with 0.42 compared to a FRS of 0.11 (p < 0.001). In accordance, individuals who experienced an event had both more often a history of heart failure and higher levels of NT-proBNP compared to those with an event-free follow-up. Evaluation of a potential association of continuous variables associated with risk, such as total cholesterol or C-reactive protein (CRP), and the selected miRs showed a weak but significant correlation of CRP with miR-378 (r = 0.22, p = 0.002) and miR-133 (r = 0.14, p = 0.048) (see Supplementary Table 2A).

**Table 1:** Baseline characteristics of the derivation study cohort (DETECT).

Derivation Cohort DETECT Study			
	Event *n* = *21*	No-Event *n* = *157*	p-value
Female, x/x(%)	21/8 (38.1)	157/93 (59.2)	0.066
Age, mean (SD)	69 (64–75)	53 (44–67)	<0.001
Cardiovascular Risk Factors			
- Arterial Hypertension, x/x(%)	21/15 (71.4)	157/57 (36.3)	0.007
- Dyslipidemia, x/x(%)	21/13 (61.9)	157/44 (28.0)	0.007
- Diabetes mellitus Type 2, x/x(%)	21/8 (38.1)	157/19 (12.1)	0.002
- Obesity (BMI > 30), x/x(%)	21/6 (28.6)	154/29 (18.8)	0.295
- Active smoker, x/x(%)	19/4 (21.1)	149/35 (23.5)	0.817
- Former smoker, x/x(%)	19/4 (21.1)	149/39 (26.2)	
- Family history of CVD, x/x(%)	21/4 (19.1)	152/23 (15.1)	0.080
History of			
- stroke, x/x(%)	21/0 (0.0)	157/0 (0.0)	—
- myocardial infarction, x/x(%)	21/1 (4.8)	157/8 (5.1)	0.948
- known coronary artery disease, x/x(%)	21/8 (38.1)	157/16 (10.2)	0.001
- known heart failure, x/x(%)	21/9 (42.9)	157/14 (8.9)	<0.001
- known PAD, x/x(%)	21/2 (9.5)	157/4 (2.6)	0.096
Laboratory parameters			
- NT-proBNP median (IQR)	152.6 (99.7–201.4)	54.8 (20.9–113.1)	<0.001
- CRP, median (IQR)	3.1 (1.5–5.2)	1.9 (0.8–4.9)	0.088
- eGFR_MDRD_, median (IQR)	49.3 (42.4–63.1)	53.4 (47.6–59.5)	0.576
- Troponin I, median (IQR)	0.011 (0.007–0.022)	0.013 (0.007–0.020)	0.859
- Total cholesterol, median (IQR)	204 (180–226)	226 (203–253)	0.010
- LDL, median (IQR)	125 (98–142)	134 (105–155)	0.159
- HDL, median (IQR)	47 (37–59)	51 (43–64)	0.196
Risk classification			
- Framingham Risk Score (FRS), median(IQR)	0.42 (0.18–0.51)	0.11 (0.06–0.28)	<0.001

### Prognostic value of selected circulating miRs and their combination in the derivation cohort

Individual levels of the selected 5 miR miRs-34a, miR-223, miR-378, miR-499 and miR-133 were not associated with the combined endpoint. All-cause 5-year mortality was associated with reduced miR-133 levels (HR: 0.09; 95% CI: 0.01, 0.86; p = 0.037) and showed a trend for the association with lower miR-223 levels (HR: 0.30; 95% CI: 0.08, 1.07; p = 0.063) (Table 2).

**Table 2:** Prognostic value of the 5 miRs and the respective Panel in the derivation study cohort (DETECT Study cohort).

		Hazard Ratio Per SD increase	95% Confidence Interval	p-value
**Mortality and/or cardiovascular events**				
n = 21 events and n = 157 controls				
miR-34a	Unadjusted	1.10	0.75; 1.62	0.622
	Adj. for age and sex	1.16	0.80; 1.66	0.435
	Adj. for age, sex and FRS	1.18	0.81; 1.72	0.381
miR-223	Unadjusted	0.45	0.11; 1.80	0.257
	Adj. for age and sex	0.87	0.65; 1.16	0.337
	Adj. for age, sex and FRS	0.82	0.60; 1.14	0.242
miR-378	Unadjusted	0.55	0.04; 6.99	0.643
	Adj. for age and sex	0.78	0.54; 1.13	0.191
	Adj. for age, sex and FRS	0.72	0.36; 1.42	0.340
miR-499	Unadjusted	0.82	0.50; 1.33	0.418
	Adj. for age and sex	1.07	0.76; 1.51	0.693
	Adj. for age, sex and FRS	1.04	0.73; 1.49	0.821
miR-133	Unadjusted	0.96	0.51; 1.79	0.886
	Adj. for age and sex	1.00	0.61; 1.66	0.991
	Adj. for age, sex and FRS	0.99	0.63; 1.54	0.958
5 miR-Panel	Unadjusted	2.57	0.77; 8.57	0.125
	Adj. for age and sex	1.21	0.44; 3.35	0.717
	Adj. for age, sex and FRS	1.40	0.51; 3.84	0.511
**Overall Mortality**				
n = 12 events and n = 166 controls				
miR-34a	Unadjusted	1.10	0.69; 1.77	0.678
	Adj. for age and sex	1.20	0.82; 1.75	0.351
	Adj. for age, sex and FRS	1.25	0.82; 1.91	0.291
miR-223	Unadjusted	0.30	0.08; 1.07	0.063
	Adj. for age and sex	0.83	0.57; 1.21	0.327
	Adj. for age, sex and FRS	0.74	0.39; 1.42	0.368
miR-378	Unadjusted	0.24	0.01; 4.14	0.327
	Adj. for age and sex	0.76	0.45; 1.29	0.308
	Adj. for age, sex and FRS	0.64	0.10; 4.22	0.646
miR-499	Unadjusted	0.88	0.48; 1.59	0.668
	Adj. for age and sex	1.26	0.84; 1.87	0.260
	Adj. for age, sex and FRS	1.21	0.79; 1.85	0.370
miR-133	Unadjusted	**0.09**	0.01; 0.86	*0*.*037*
	Adj. for age and sex	**0.15**	0.03; 0.81	*0*.*028*
	Adj. for age, sex and FRS	**0.16**	0.03; 0.96	*0*.*046*
5 miR-Panel	Unadjusted	**3.03**	1.09; 8.43	*0*.*034*
	Adj. for age and sex	**2.16**	1.10; 4.24	*0*.*025*
	Adj. for age, sex and FRS	**2.26**	1.02; 6.13	*0*.*031*

Combination of the 5 miRs as panel showed a robust association with overall mortality with HR of 3.03 (95% CI: 1.09, 8.43; p = 0.034) in an unadjusted model and of 2.89 (95% CI: 1.32, 6.33; p = 0.008) after adjustment for the FRS variables (Table 2) or for the biomarkers NT-proBNP or troponin I (Supplementary Table 3). Nevertheless, no significant association with the combined endpoint was observed (Tables 2 and 3).

Furthermore, the miR panel significantly improved the discriminatory value of the FRS variables to identify patients at risk to die within 5 years with an increase of the area under the curve in the receiver operator characteristic analysis from 0.77 to 0.85 (p = 0.039)^32^. The respective c-index tended to be improved from 0.73 to 0.85 (p = 0.074).

### Validation cohort, characteristics and differences to the derivation cohort

Of the 129 individuals of the SHIP validation cohort, 64 died and 43 experienced a cardiovascular event within the 12-year follow-up period. In this primary prevention population, the proportion of traditional risk factors did not differ between individuals who died and survivors. The respective baseline characteristics classified according to event status are given as Table 3.

**Table 3:** Baseline characteristics of the validation study cohort (SHIP).

Validation Cohort SHIP Study			
	Event *n* = *64*	No-Event *n* = *65*	p-value
Female, x/x(%)	26/38 (40.63%)	28/37 (43.08%)	0.778
Age, mean (SD)	75.86 (7.74)	73.60 (8.68)	0.795
Cardiovascular Risk Factors			
- Arterial Hypertension, x/x (%)	57/7 (89.06%)	59/6 (90.77%)	0.747
- Dyslipidemia, x/x (%)	36/28 (56.25%)	35/30 (53.85%)	0.784
- Diabetes mellitus Type 2, x/x (%)	34/30 (53.13%)	23/42 (35.38%)	0.042
- Obesity (BMI > 30), x/x (%)	36/28 (56.25%)	33/32 (50.77%)	0.533
- Active smoker, x/x (%)	4/60 (6.25%)	4/61 (6.15%)	0.982
- Former smoker, x/x (%)	35/29 (54.69%)	30/35 (46.15%)	0.332
History of			
- stroke, x/x(%)	—	—	—
- myocardial infarction, x/x (%)	—	—	—
- known coronary artery disease, x/x (%)	—	—	—
- known heart failure, x/x (%)	—	—	—
- known PAD, x/x (%)	—	—	—
Laboratory parameters			
- CRP, median (IQR)	2.97 (1.22–6.37)	1.62 (0.77–3.65)	0.011
- eGFR_MDRD_, median (IQR)	64.48 (50.67–86.03)	62.24 (52.65–84.07)	0.660
Risk classification			
- Framingham Risk Score (FRS), median (IQR)	0.19 (0.10–0.35)	0.17 (0.05–0.34)	0.596
Systematic COronary Risk Evaluation (SCORE), median (IQR)	0.11 (0.05–0.17)	0.09 (0.06–0.15)	0.377

In this population-based sample, the associations of CRP with miR-378 and miR-133 observed in the derivation cohort could not be replicated; furthermore a weak negative correlation of CRP and miR-499 was seen (r = −0.20, p = 0.03) (Supplementary Table 2B).

### Prognostication using circulating miRs in the validation cohort

The predictive information of the prognostic miR panel observed in the derivation cohort could be confirmed in the validation cohort with a HR of 1.31 (95% CI: 1.03, 1.66; p = 0.03) to predict all-cause mortality after adjusting for the FRS variables. Using cardiovascular events as outcome measure, application of the prognostic miR panel resulted in a HR of 1.29 (95% CI: 0.99, 1.69; p = 0.0) in a FRS adjusted model. Comparable results are seen if SCORE instead of FRS variables were used for adjustment (Table 4).

**Table 4:** Prognostic value of the 5 miR Panel in the validation study cohort (SHIP).

		Hazard Ratio per SD increase	95% Confidence Interval	p-value
**Mortality**				
n = 64 events and n = 65 controls				
5 miR-Panel	Adjusted for age and sex	1.32	1.04–1.68	*0*.*02*
	Adjusted for Framingham Risk Score variables	1.31	1.03–1.66	*0*.*03*
	Adjusted for SCORE variables	1.29	1.02–1.64	*0*.*03*
**Cardiovascular events**				
n = 43 events and n = 86 controls				
5 miR-Panel	Adjusted for age and sex	1.33	1.00–1.77	0.05
	Adjusted for Framingham Risk Score variables	1.29	0.99–1.69	0.06
	Adjusted for SCORE variables	1.29	0.98–1.68	0.07

In order to more closely reflect a potential real world application, the prognostic miR panel was further evaluated in a dichotomized fashion, providing the information high vs. low mortality risk. Here, the miR panel showed a FRS independent association with mortality and cardiovascular events with HR of 1.31 (95% CI: 1.03, 1.66; p = 0.03) and of 1.29 (95% CI: 0.99, 1.69; p = 0.06), respectively (Supplementary Table 4). Of interest, none of the 5 miRs showed a significant association with cardiovascular events or mortality, if analyzed individually (Supplementary Table 5). As sensitivity analysis, all possible 4 marker combinations were calculated yielding HRs from 1.13 to 1.33 for the prediction of death (Supplementary Table 6).

To further evaluate, if the prognostic miR panel provides information beyond that provided by the established risk scores such as the FRS or SCORE, reclassification analyses were performed. Here, a risk stratification model based on the FRS or the SCORE variables was significantly improved regarding the prediction of mortality by addition of the 5 miR panel. This is substantiated by a continuous net reclassification index (cNRI) of 0.42 (p = 0.014) and 0.49 (p = 0.005) as well as an integrated discrimination improvement (IDI) of 0.03 (p = 0.047) and 0.027 (p = 0.065) of a base model using FRS or SCORE variables, respectively, (Table 5). Nevertheless, the miR panel was not able to improve the c-index of a model based on the FRS or the SCORE variables (Data not shown).

**Table 5:** Risk reclassification in the validation cohort based on a base model of the Framingham Risk Score (FRS) variables (A) or the SCORE system of the European society of cardiology (B) amended by the 5 miR Panel.

	Continuous NRI	p-Value	IDI	Relative IDI	p-Value
**A – Base model FRS**					
Mortality n = 64 events and n = 65 controls	0.42	0.014	0.03	75%	0.047
Cardiovascular events n = 43 events and n = 86 controls	0.35	0.055	0.021	67%	0.14
**B – Base model SCORE**					
Mortality n = 64 events and n = 65 controls	0.49	0.005	0.027	82%	0.065
Cardiovascular events n = 43 events and n = 86 controls	0.33	0.073	0.021	68%	0.15

NRI denotes net reclassification improvement and IDI denotes integrated discrimination improvement.

## Discussion

The results of the present study suggest, for the first time, that a panel of specific circulating miRs, measured from peripheral venous blood samples, may provide valid prognostic information in the context of risk prediction in prevention.

The proposed panel includes 5 distinct miRs that represent different pathomechanisms, namely age, inflammation, metabolism, and muscle damage that are known to play important roles in cardiovascular disease development and progression.

If analyzing the miRs individually, not as panel, the selected miR did not show a reproducible association with outcome. In contrast, the combined use of the 5 miRs as a panel showed a robust prediction of mortality in the derivation and validation cohort independently of the clinical risk variables. Furthermore, the miR panel significantly improved the prognostic value of e.g. the FRS variables to identify patients who will die with e.g. an AUC improvement from of 0.77 to 0.85 (p = 0.039) in the derivation cohort and a cNRI of 0.42 (p = 0.014) in the validation cohort. Within the validation cohort the prognostic power of our panel provided a lower HR compared to the validation cohort. As the validation cohort represents patients at risk whereas the validation study stems from a population based cohort with low risk this difference is what one would expect.

There are very few data on the potential use of miRs as biomarkers for outcome prediction in cardiovascular prevention. Zampetaki *et al*. evaluated 19 candidate miRs in a population based cohort of 820 individuals with respect to their relation with incident myocardial infarction. Here, a significant association of miR-126, miR-223 and miR-197 was observed^33^. Moreover, this study revealed that platelets are a major contributor to circulating levels of miR-126.

Furthermore, an association of circulating miR levels with clinical outcome could be shown in studies investigating patients with acute coronary syndrome. In those studies, the muscle-enriched miR-133a^34, 35^ and miR-499^36, 37^ were suggested to provide prognostic information, although the added value to the classical myocardial damage marker troponin was only marginal.

Conventional biomarkers associated with inflammation such as C-reactive protein^38^, with left ventricular wall stress such as natriuretic peptides^39^ or with myocardial damage such as cardiac troponins^40, 41^ have shown prognostic value in general population cohorts. Furthermore, the concept of using a combination of markers picturing different pathophysiologic mechanisms of cardiovascular disease development and progression has been proposed to improve risk estimation if added to conventional risk models as used in daily clinical routine^9, 42, 43^.

Use of miR signatures provides superior diagnostic information compared to a single miR^44^ in evaluation of acute myocardial infarction supporting the concept of using a miR panel rather than a specific miR that might reflect only one aspect of cardiovascular disease development.

Given the multifunctional mechanisms involved in cardiovascular disease development and mortality, it is not surprising that combining analytes of different biomarkers reflecting different underlying pathophysiological mechanisms will therefore provide superior information as also exemplified in the present study for circulating miRs.

### Limitations

The most important limitation of the presents study relates to the limited number of endpoints in our case-control cohorts. It is one of the first studies evaluating the use of a miR panel for risk prediction; given this limitation, a prospectively and independently validation of the observed results is needed. Moreover, the used risk scores were developed mainly to predict cardiovascular events in population-based cohorts. In the present study, while the miR panel robustly predicted overall mortality, the prognostic value for cardiovascular endpoints was less convincing.

## Conclusion

A panel of 5 selected circulating miRs associated with distinctive pathophysiological mechanisms is able to improve risk stratification in primary and secondary prevention with respect to mortality beyond and amendatory to the widely-used Framingham Risk Score.

## Electronic supplementary material

Supplementary dataset
