# Supplementary material for: Improved risk stratification in prevention by use of a panel of selected circulating microRNAs
Source: Sci Rep. 2017 Jul 3;7:4511. doi: 10.1038/s41598-017-04040-w (PMC5495799; doi:10.1038/s41598-017-04040-w)
Supplement: Supplementary file 1 — Supplementary dataset [file 41598_2017_4040_MOESM1_ESM.doc]

**Supplementary Information for:**

**Improved risk stratification in prevention**

**by use of a panel of selected circulating microRNAs**

Till Keller, MD*^1,2,16^; Jes-Niels Boeckel, PhD*^1,2,3^; Stefan Groß, PhD^5,2^; Jens Klotsche, PhD^4^; Lars Palapies, MSc^1^; David Leistner, MD^1,14^; Lars Pieper, PhD^4^; Günnter K Stalla, MD^6^;

Hendrik Lehnert, MD^7^; Sigmund Silber, MD^8^; David Pittrow, MD^11^; Winfried Maerz, MD^9^;

Marcus Dörr, MD^5,15^; Hans-Ulrich Wittchen, MD^4^; Sebastian E. Baumeister, PhD^12,13^;

Uwe Völker, PhD^10^; Stephan B Felix, MD^5,15^; Stefanie Dimmeler, PhD*^2,3^; Andreas M Zeiher, MD*^1,2^

^1^ Department of Internal Medicine III, Cardiology, University Hospital, Goethe University Frankfurt, Frankfurt, Germany

^2^ German Center for Cardiovascular Disease (DZHK), Partnersite RheinMain

^3^ Institute of Cardiovascular Regeneration, Centre for Molecular Medicine, Goethe University Frankfurt, Frankfurt, Germany

^4^ Klinische Psychologie und Psychotherapie, Technical University Dresden, Dresden, Germany

^5^ Innere Klinik B, Universitätsmedizin Greifswald, Greifswald, Germany

^6^ Max­Plank­Institut für Psychiatrie, Neuroendokrinologie, München, München, Germany

^7^ Medizinische Klinik I, Universitätsklinikum Schleswig­Holstein, Lübeck, Germany

^8^ Praxisklinik, Kardiologische Gemeinschaftspraxis, München, Germany

^9^ synlab Akademie für ärztliche Fortbildung, Synlab Services GmbH, Mannheim, Germany

^10^ Interfakultäres Institut für Genetik & Funktionelle Genomforschung, Universitätsmedizin Greifswald, Greifswald, Germany

^11^ Institute of Clinical Pharmacology, Technical University Dresden, Dresden, Germany

^12^ Institute for Community Medicine, University Medicine Greifswald, Germany

^13^ Institute of Epidemiology and Preventive Medicine, University of Regensburg, Germany

^14^ German Center for Cardiovascular Disease (DZHK), Partnersite Berlin

^15^ German Center for Cardiovascular Disease (DZHK), Partnersite Greifswald

^16^ Department of Cardiology, Kerckhoff Heart and Thorax Centre, Bad Nauheim, Germany

* Authors contributed equally as first and senior authors

| **Derivation Cohort**  **DETECT Study** | | |
| --- | --- | --- |
|  | **Derivation Cohort**  *n=178* | **Total DETECT Cohort**  (only patients without renal failure, CHD anf heart failure)  *n=5691* |
| Female, x/x(%) | 178 / 101 (56.7) | 5,691 / 3,536 (62.1) |
| Age, mean (SD) | 54 (45 - 68) | 56 (45 - 67) |
| CVRF |  |  |
| - Arterial Hypertension, x/x(%) | 178 / 72 (40.5) | 5,691 / 2,026 (35.6) |
| - Dyslipidaemia, x/x(%) | 178 / 57 (32.0) | 5,691 / 1,764 (31.0) |
| - Diabetes mellitus Type 2, x/x(%) | 178 / 27 (15.2) | 5,691 / 810 (14.2) |
| - Obesity (BMI>30), x/x(%) | 175 / 35 (20.0) | 5,617 / 1,300 (23.1) |
| - Active smoker, x/x(%) | 168 / 39 (23.2) | 5,493 / 1,209 (22.0) |
| - Former smoker, x/x(%) | 168 / 43 (25.6) | 5,493 / 1,191 (21.7) |
| - Family history of CVD, x/x(%) | 173 / 27 (15.6) | 5,496 / 825 (15.0) |
| History |  |  |
| - of stroke, x/x(%) | 178 / 0 (0.0) | 5,691 / 74 (1.3) |
| - of myocardial infarction, x/x(%) | 178 / 9 (5.1) | 5,691 / 11 (0.2) |
| - known coronary artery disease, x/x(%) | 178 / 24 (13.5) | 5,691 / 34 (0.6) |
| - known heart failure, x/x(%) | 178 / 23 (12.9) | 5,691 / 121 (2.1) |
| - known PAD, x/x(%) | 178 / 6 (3.4) | 5,691 / 115 (2.0) |
| Laboratory parameters |  |  |
| - NT-proBNP median(IQR) | 71.4 (23.2 - 120.6) | 58.2 (29.2 - 117.6) |
| - CRP, median(IQR) | 2.1 (0.9 - 4.9) | 2.1 (1.0 - 4.5) |
| - eGFR_MDRD_, median(IQR) | 54.0 (47.4 - 60.3) | 57.6 (50.5 - 64.8) |
| - Troponin I, median(IQR) | 0.011 (0.007 - 0.020) | 0.009 (0.007 - 0.014) |
| - Total cholesterol, median(IQR) | 224 (200 - 256) | 223 (197 - 252) |
| - LDL, median(IQR) | 133 (103 - 154) | 128 (106 - 150) |
| - HDL, median(IQR) | 51 (42 - 64) | 53 (43 - 66) |
| Risk classification |  |  |
| - Framingham Risk Score (FRS), median(IQR) | 6 (2 - 17) | 6 (2 - 17) |

**Supplementary Table 1:** Baseline characteristics of the derivation cohort based on the DETECT study in comparison to the overall DETECT study cohort of patients without prevalent renal or cardiac disease

| **A - DETECT Study cohort** | | **miR-34a** | **miR-223** | **miR-378** | **miR-499** | **miR-133** |
| --- | --- | --- | --- | --- | --- | --- |
| Total cholesterol | Corr.coef. | 0.06 | 0.07 | 0.02 | 0.06 | 0.07 |
|  | p-value | 0.420 | 0.333 | 0.812 | 0.375 | 0.353 |
| eGFR_MDRD_ | Corr.coef. | 0.01 | 0.00 | 0.09 | 0.01 | -0.05 |
|  | p-value | 0.927 | 0.967 | 0.218 | 0.850 | 0.511 |
| CRP | Corr.coef. | -0.01 | 0.07 | 0.22 | 0.03 | 0.14 |
|  | p-value | 0.916 | 0.351 | 0.002 | 0.730 | 0.048 |

| **B - SHIP Study cohort** | | **miR-34a** | **miR-223** | **miR-378** | **miR-499** | **miR-133** |
| --- | --- | --- | --- | --- | --- | --- |
| Total cholesterol | Corr.coef. | - 0.080 | 0.020 | 0.044 | - 0.072 | - 0.024 |
|  | p-value | 0.37 | 0.82 | 0.62 | 0.42 | 0.78 |
| eGFR_MDRD_ | Corr.coef. | 0.027 | 0.12 | 0.058 | 0.13 | 0.063 |
|  | p-value | 0.76 | 0.19 | 0.51 | 0.14 | 0.48 |
| CRP | Corr.coef. | 0.10 | 0.10 | 0.16 | - 0.20 | 0.000 |
|  | p-value | 0.24 | 0.26 | 0.08 | *0.03* | 1 |

**Supplementary Table 2:** Correlation of the evaluated miRs with potential confounders in the derivation cohort (DETECT Study cohort) (A) and in the validation cohort (SHIP Study cohort) (B)

|  |  | **Hazard Ratio**  **Per SD increase** | **95% Confidence Interval** | **p-value** |
| --- | --- | --- | --- | --- |
| **Mortality and/or cardiovascular events**  n=21 events and n=157 controls | | | | |
| miR-34a | Unadjusted | 1.10 | 0.75 ; 1.62 | 0.622 |
|  | Adj. for age, sex and NT-proBNP | 1.37 | 0.94 ; 1.99 | 0.101 |
|  | Adj. for age, sex and Troponin I | 1.21 | 0.85 ; 1.72 | 0.298 |
|  | Adj. for age, sex and CRP | 1.20 | 0.83 ; 1.73 | 0.340 |
| miR-223 | Unadjusted | 0.45 | 0.11 ; 1.80 | 0.257 |
|  | Adj. for age, sex and NT-proBNP | 0.83 | 0.64 ; 1.08 | 0.168 |
|  | Adj. for age, sex and Troponin I | **0.72** | 0.57 ; 0.91 | *0.007* |
|  | Adj. for age, sex and CRP | 0.71 | 0.32 ; 1.57 | 0.398 |
| miR-378 | Unadjusted | 0.55 | 0.04 ; 6.99 | 0.643 |
|  | Adj. for age, sex and NT-proBNP | 0.75 | 0.53 ; 1.05 | 0.094 |
|  | Adj. for age, sex and Troponin I | **0.66** | 0.50 ; 0.86 | *0.002* |
|  | Adj. for age, sex and CRP | 0.45 | 0.02 ; 9.13 | 0.600 |
| miR-499 | Unadjusted | 0.82 | 0.50 ; 1.33 | 0.418 |
|  | Adj. for age, sex and NT-proBNP | 1.08 | 0.76 ; 1.52 | 0.674 |
|  | Adj. for age, sex and Troponin I | 1.07 | 0.75 ; 1.53 | 0.719 |
|  | Adj. for age, sex and CRP | 1.09 | 0.77 ; 1.54 | 0.629 |
| miR-133 | Unadjusted | 0.96 | 0.51 ; 1.79 | 0.886 |
|  | Adj. for age, sex and NT-proBNP | 0.99 | 0.58 ; 1.68 | 0.974 |
|  | Adj. for age, sex and Troponin I | 0.84 | 0.30 ; 2.33 | 0.740 |
|  | Adj. for age, sex and CRP | 0.97 | 0.53 ; 1.78 | 0.928 |
| 5 miR-Panel | Unadjusted | 2.57 | 0.77 ; 8.57 | 0.125 |
|  | Adj. for age, sex and NT-proBNP | 1.56 | 0.47 ; 5.21 | 0.471 |
|  | Adj. for age, sex and Troponin I | 2.13 | 0.79 ; 5.75 | 0.136 |
|  | Adj. for age, sex and CRP | 1.80 | 0.64 ; 5.06 | 0.262 |
| miR-Panel | Unadjusted | 2.57 | 0.77 ; 8.57 | 0.125 |
| **Overall Mortality**  n=12 events and n=166 controls | | | | |
| miR-34a | Unadjusted | 1.10 | 0.69 ; 1.77 | 0.678 |
|  | Adj. for age, sex and NT-proBNP | 1.43 | 0.96 ; 2.13 | 0.078 |
|  | Adj. for age, sex and Troponin I | 1.20 | 0.82 ; 1.75 | 0.357 |
|  | Adj. for age, sex and CRP | 1.19 | 0.82 ; 1.74 | 0.363 |
| miR-223 | Unadjusted | 0.30 | 0.08 ; 1.07 | 0.063 |
|  | Adj. for age, sex and NT-proBNP | 0.80 | 0.61 ; 1.06 | 0.118 |
|  | Adj. for age, sex and Troponin I | 0.80 | 0.56 ; 1.14 | 0.213 |
|  | Adj. for age, sex and CRP | 0.84 | 0.58 ; 1.23 | 0.373 |
| miR-378 | Unadjusted | 0.24 | 0.01 ; 4.14 | 0.327 |
|  | Adj. for age, sex and NT-proBNP | 0.74 | 0.52 ; 1.06 | 0.096 |
|  | Adj. for age, sex and Troponin I | 0.73 | 0.46 ; 1.16 | 0.185 |
|  | Adj. for age, sex and CRP | 0.78 | 0.51 ; 1.19 | 0.252 |
| miR-499 | Unadjusted | 0.88 | 0.48 ; 1.59 | 0.668 |
|  | Adj. for age, sex and NT-proBNP | 1.28 | 0.87 ; 1.90 | 0.215 |
|  | Adj. for age, sex and Troponin I | 1.25 | 0.84 ; 1.86 | 0.261 |
|  | Adj. for age, sex and CRP | 1.25 | 0.84 ; 1.85 | 0.267 |
| miR-133 | Unadjusted | **0.09** | 0.01 ; 0.86 | *0.037* |
|  | Adj. for age, sex and NT-proBNP | **0.20** | 0.05 ; 0.76 | *0.018* |
|  | Adj. for age, sex and Troponin I | **0.15** | 0.03 ; 0.81 | *0.027* |
|  | Adj. for age, sex and CRP | 0.14 | 0.02 ; 1.15 | 0.068 |
| 5 miR-Panel | Unadjusted | **3.03** | 1.09 ; 8.43 | *0.034* |
|  | Adj. for age, sex and NT-proBNP | **2.10** | 1.09 ; 4.05 | *0.026* |
|  | Adj. for age, sex and Troponin I | **2.19** | 1.10 ; 4.38 | *0.026* |
|  | Adj. for age, sex and CRP | 2.19 | 0.99 ; 4.82 | 0.052 |
|  |  |  |  |  |

**Supplementary Table 3:** Prognostic information of the 5 miRs individually and the respective 5 miR Panel in the derivation cohort (DETECT Study cohort) with adjustment for established biomarkers as potential confounders.

|  |  | **Hazard Ratio** | **95% Confidence Interval** | **p-value** |
| --- | --- | --- | --- | --- |
| **Mortality**  n=64 events and n=65 controls | | | | |
| 5 miR-Panel | Adjusted for age and sex | 1.32 | 1.04 – 1.68 | *0.02* |
|  | Adjusted for Framingham Risk Score variables | 1.31 | 1.03 – 1.66 | *0.03* |
| **Cardiovascular events**  n=43 events and n=86 controls | | | | |
| 5 miR-Panel | Adjusted for age and sex | 1.33 | 1.00 – 1.77 | *0.05* |
|  | Adjusted for Framingham Risk Score variables | 1.29 | 0.99 – 1.69 | *0.06* |
|  |  |  |  |  |

**Supplementary table 4:** Prognostic information of the 5 miR Panel in the validation cohort (SHIP Study) after dichotomization according to an individually optimized threshold obtained by maximizing the Youden index in receiver operator characteristics analyses.

|  |  | **Hazard Ratio**  **Per SD increase** | **95% Confidence Interval** | **p-value** |
| --- | --- | --- | --- | --- |
| **Mortality**  n=64 events and n=65 controls | | | | |
| miR-34a | Adj. for age and sex | 1.07 | 0.85 - 1.35 | 0.56 |
|  | Adj. for age, sex and FRS variables | 1.07 | 0.84 - 1.38 | 0.57 |
| miR-223 | Adj. for age and sex | 1.00 | 0.77 - 1.28 | 0.98 |
|  | Adj. for age, sex and FRS variables | 0.99 | 0.76 - 1.27 | 0.91 |
| miR-378 | Adj. for age and sex | 1.12 | 0.90 - 1.39 | 0.32 |
|  | Adj. for age, sex and FRS variables | 1.09 | 0.88 - 1.36 | 0.41 |
| miR-499 | Adj. for age and sex | 0.98 | 0.77 - 1.24 | 0.84 |
|  | Adj. for age, sex and FRS variables | 0.98 | 0.77 - 1.24 | 0.84 |
| miR-133 | Adj. for age and sex | 1.15 | 0.92 - 1.39 | 0.24 |
|  | Adj. for age, sex and FRS variables | 1.15 | 0.94 - 1.41 | 0.19 |
| **Cardiovascular events**  n=43 events and n=86 controls | | | | |
| miR-34a | Adj. for age and sex | 1.06 | 0.80 - 1.40 | 0.69 |
|  | Adj. for age, sex and FRS variables | 1.05 | 0.78 - 1.41 | 0.75 |
| miR-223 | Adj. for age and sex | 1.02 | 0.75 - 1.37 | 0.92 |
|  | Adj. for age, sex and FRS variables | 0.99 | 0.73 - 1.35 | 0.95 |
| miR-378 | Adj. for age and sex | 1.14 | 0.87 - 1.49 | 0.35 |
|  | Adj. for age, sex and FRS variables | 1.12 | 0.86 - 1.47 | 0.41 |
| miR-499 | Adj. for age and sex | 0.98 | 0.73 - 1.31 | 0.88 |
|  | Adj. for age, sex and FRS variables | 0.99 | 0.73 - 1.33 | 0.93 |
| miR-133 | Adj. for age and sex | 1.16 | 0.91 - 1.48 | 0.22 |
|  | Adj. for age, sex and FRS variables | 1.15 | 0.90 - 1.47 | 0.27 |
|  |  |  |  |  |

**Supplementary Table 5:** Prognostic value of the 5 individual miRs in the validation cohort

|  |  | **Hazard Ratio**  **per SD increase** | **95% Confidence Interval** | **p-value** |
| --- | --- | --- | --- | --- |
| **Mortality**  n=64 events and n=65 controls | | | | |
| 5 miR-Panel |  | 1.32 | 1.04 - 1.68 | *0.02* |
| 4 miR Panel 1 | mir223, mir378, mir499, mir133a | 1.33 | 1.05 - 1.68 | *0.02* |
| 4 miR Panel 2 | mir34a, mir378, mir499, mir133a | 1.13 | 0.89 - 1.43 | 0.32 |
| 4 miR Panel 3 | mir34a, mir223, mir499, mir133a | 1.23 | 1.00 - 1.53 | 0.05 |
| 4 miR Panel 4 | mir34a, mir223, mir378, mir133a | 1.30 | 1.03 - 1.66 | *0.03* |
| 4 miR Panel 5 | mir34a, mir223, mir378, mir499 | 1.16 | 0.90 - 1.49 | 0.26 |
| **Cardiovascular events**  n=43 events and n=86 controls | | | | |
| 5 miR-Panel |  | 1.33 | 1.00 - 1.77 | 0.05 |
| 4 miR Panel 1 | mir223, mir378, mir499, mir133a | 1.34 | 1.02 - 1.76 | 0.04 |
| 4 miR Panel 2 | mir34a, mir378, mir499, mir133a | 1.17 | 0.88 - 1.54 | 0.28 |
| 4 miR Panel 3 | mir34a, mir223, mir499, mir133a | 1.23 | 0.97 - 1.56 | 0.09 |
| 4 miR Panel 4 | mir34a, mir223, mir378, mir133a | 1.32 | 0.99 - 1.76 | 0.06 |
| 4 miR Panel 5 | mir34a, mir223, mir378, mir499 | 1.17 | 0.87 - 1.58 | 0.30 |
|  |  |  |  |  |

**Supplementary Table 6:** Sensitivity Analysis of the Prognostic value of the miR Panel in the validation study cohort (SHIP) by calculating the Hazard Ratios for all possible 4 miR marker combinations. All Hazard ratios are adjusted for age and sex.
